# Sensitivity of DiaRem Scoring System in Predicting Type Two Diabetes Mellitus Resolution After Bariatric Surgery in Qassim Region

**DOI:** 10.7759/cureus.20064

**Published:** 2021-11-30

**Authors:** Bandar Saad Assakran, Mansur S Alqunai, Abdullah Homood Alromaih, Laila M Almutairi, Fai M Alharbi, Leen M Almaghyuli

**Affiliations:** 1 Department of Surgery, King Fahad Specialist Hospital, Buraydah, SAU; 2 Department of Surgery, College of Medicine, Jouf University, Sakaka, SAU; 3 General Surgery, Qassim University, Buraydah, SAU

**Keywords:** type two diabets mellitus, diabetes remission, diabetic patients, bariatric surgery, diarem

## Abstract

Introduction: Type two diabetes mellitus (T2DM) remission has been observed as an additional benefit of bariatric surgery for morbidly obese diabetic patients. There are many scoring systems for identifying factors that predict diabetes remission; however, there is as yet no universally applicable scoring system.

Aim: This study aims to test the sensitivity of the DiaRem scoring system for predicting the resolution of T2DM in morbidly obese patients who underwent bariatric surgery at King Fahad Specialist Hospital in Buraydah, Saudi Arabia.

Methods: This was a non-randomized controlled trial conducted at King Fahd Specialist Hospital in Buraydah, Saudi Arabia. Visiting patients at first screening were enrolled based on eligibility criteria. Data were collected according to the given parameters such as gender, age, body mass index (BMI), duration of diabetes mellitus (DM), medications (insulin, oral antihyperglycemic agents, number of tablets if used, or no medications use), presence of comorbidities, such as hypertension and dyslipidemia, HbA1c level (before surgery and at third, sixth, and 12th months after surgery), and fasting blood glucose (FBG) level (before and after surgery).

Results: A total of 96 diabetic patients were enrolled (35 males vs 61 females) with a mean age of 46.5 years. Laparoscopic sleeve gastrectomy was the most commonly performed surgery. The most common associated comorbidities were hypertension (50%) and hypothyroidism (14.6%). Results of the DiaRem scoring system showed 0-2 points in 15.6% patients, 3-7 points in 39.6% patients, 8-12 in 26% patients, 13-17 in 9.4% patients, and 18-22 in 9.4% patients. The lowest DiaRem score was associated with a higher value of BMI, shorter DM duration, and lower mean values of HbA1c and FBG post-surgery.

Conclusion: Consistent with the literature, our results indicated that those with an increased BMI, shorter duration of DM, and lower values of HbA1c post-FBG had a greater chance of diabetes remission postoperatively.

## Introduction

Obesity is a metabolic disease that can be clinically defined as a body mass index (BMI) >30 kg/m^2^. The World Health Organization (WHO) stated that in 2016, there were 650 million obese people worldwide [1]. In the same year, in Saudi Arabia, the obesity rate was 34.7% [2]. Obesity is linked to the development of type two diabetes mellitus (T2DM) [3]. Many studies have been done in Saudi Arabia to identify the prevalence of T2DM in obese patients. Diabetes mellitus (DM) was considerably higher among obese patients, as indicated by Alqubali et al.’s study on 549 obese and overweight patients: 22.8% were diabetic [4].

The work by Pories et al. led to the assumption that T2DM is surgically curable in morbidly obese diabetic patients [5]. In 2009, the American Diabetes Association included bariatric surgery as a component of the management of patients living with obesity and diabetes [6]. In 2011, the International Diabetes Federation also acknowledged bariatric surgery as part of the management of diabetic obese patients [7].

Although the original purpose of bariatric surgery was limited to weight reduction, recent studies have shown the great effect of bariatric surgery on the remission of T2DM in patients with obesity [8]. A meta-analytic study of 621 articles that studied the impact of bariatric surgery on type two diabetes showed that 78.1% of diabetic patients had total resolution after gastric bypass surgery, and diabetes was improved or, in some cases, resolved in 86.6% of patients [9]. In another meta-analysis of nine articles with 343 participants, 269 patients were included; 90.9%-95.7% of patients had remission of T2DM and achieved HbA1c < 7.0% [10]. Park et al. studied 621 patients who underwent Roux-en-Y gastric bypass (RYGB); 110 were diagnosed with T2DM, of whom 68.4% showed complete remission of T2DM [11]. These meta-analyses show the important role of bariatric surgery in the remission of T2DM in obese patients. A retrospective study in Saudi Arabia was carried out on 318 obese patients who underwent either LSG or RYGB, 58 of whom were diabetic. This study aimed to measure the percentage of HbA1c reduction after bariatric surgery on the two groups; the authors found that the target of HbA1c 6.5% or less was observed in 62.5% of diabetic patients and 97.1% of the non-diabetic patients [12]. Another study was done in Saudi Arabia on 56 morbidly obese patients with T2DM; a follow-up of 30 months after LSG showed a resolution of diabetes in 22%, 54%, and 90% in the third, sixth, and 12th months postoperatively, respectively [13]. Buchwald et al. reported that the rate of diabetes remission was 80.3% with RYGB, 95.1% with biliopancreatic diversion (BPD), and 56.7% with laparoscopic adjustable gastric banding [9].

The vast majority of patients with obesity and T2DM will attain remission of T2DM by six weeks postoperatively [14]. Other studies showed immediate improvement or reduction of hyperglycemia after bariatric surgery in morbidly obese diabetic patients. In conclusion, bariatric surgery has shown that it has been more effective in achieving remission of T2DM than conventional therapy [8,15].

Remission of diabetes

The remission of diabetes has been defined in several ways in the literature; consequently, the remission rate of diabetes can be greatly affected by the choice of definition [16]. This can be evident by Blackstone et al., who suggested five different models to define DM remission, which varied from 43.2% to 59.4% according to the predictors that have been used [17]. Buchwald et al. defined resolution of diabetes by the percentage of patients who were off antidiabetics, with normal fasting blood glucose (FBG), and with glycated hemoglobin less than 6%, which have been achieved by 80.5% of patients [9]. Most studies define diabetes remission according to the level of HbA1c (<6%) and discontinuation of using antidiabetes medications [10,11,18,19].

Predictors of diabetes remission

In most studies, preoperative parameters, such as age, diabetes duration, insulin use, baseline BMI, level of fasting glucose, and HbA1c, are predictors of DM remission [20]. These predictors can be divided into two groups, predictors that can indicate preserved pancreatic beta-cell function (including C-peptide level, duration of DM, age, and glycemic control) and predictors that signify the decrease of insulin resistance (including BMI, visceral fat, and weight reduction after bariatric surgery) [16]. The preoperative predictors of DM remission include clinical (age, BMI, duration of DM, and severity of DM) and biochemical (FBG and HbA1c) predictors.

Clinical Predictors

Age: Although age represents the well-being of the patients and the general physiological function, it can also predict the pancreatic beta-cell preserve. Thus, pancreatic beta-cell mass and function decline with increasing age [21]. A meta-analysis of 13 studies by Wang et al. showed a negative association between age and the remission of T2DM. Therefore, younger patients with T2DM may have a higher remission rate [20]. This is evident from Hamza et al., who found that in each additional 12 years of age, the T2DM remission rate will be reduced by 20% [22].

BMI: Lee et al.'s study was the first to identify the relation between low BMI value and the rate of T2DM remission, which was proportional to each other; patients with low BMI value will have a lower rate of diabetes remission [23]. This was similarly reported by Scopinaro et al., a study that stated that the remission rate of diabetes would be increased with the increase of preoperative BMI values [10]. In a study on 134 morbidly obese patients with T2DM who underwent laparoscopic RYGB, diabetes remission was significant in young patients with high BMI values [11].

Duration of DM: Pories et al.'s study was the first to report the importance of T2DM duration concerning diabetes remission; he found that those who failed to retrieve a normal blood glucose control were those having a longer diabetes duration [5]. Panunzi et al. studied the predictors of diabetes remission in 415 patients; shorter T2DM duration and lower baseline glycemia were significantly associated with a higher remission rate [24]. Rosenthal et al. conducted a retrospective review on diabetic patients who underwent laparoscopic sleeve gastrectomy. The patients were divided into two groups according to the duration of diabetes, more than five years and less than five years. All participants showed diabetes clinical resolution or improvement, but it was significant in patients with a short duration of diabetes [25]. Similarly, in a meta-analysis by Wang et al. (13 studies including 1555 patients with T2DM), the remission rate was significantly associated with a shorter diabetes duration [20].

The severity of DM: The severity of diabetes might be reflected by antidiabetic agents or insulin to control hyperglycemia [26]. In Hayes’s decision tree to predict the resolution after one year, he studied 127 patients with T2DM and obesity who underwent gastric bypass. Thirty-two of the participants required insulin to control hyperglycemia, half of them remained diabetic, and six of those continued to use insulin after one year following the surgery. Consequently, patients taking insulin prior to bariatric surgery are skeptical about having diabetes resolution [19]. Correspondingly, in a prospective observational study done on 106 obese diabetics, the authors reported a higher remission rate in patients who were preoperatively not on insulin for glycemic control [27]. In a retrospective analysis study of Blackstone et al., on 505 patients, he observed that the remission rates were higher for patients not using insulin preoperatively in comparison to the opposite group (53.8% vs 13.5%) [17].

Biochemical Predictors

HbA1c: Glycated hemoglobin (HbA1c) reflects the range of plasma glucose fluctuation over the past eight to 12 weeks [27]. A lower plasma level of HbA1c is associated with a higher possibility of T2DM remission following bariatric surgery [26]. A meta-analysis of 13 studies included 1149 T2DM patients and aimed to study the relationship between preoperative HbA1c level and diabetes remission following bariatric surgery. Patients with increased baseline HbA1c levels were least likely to experience resolution of their diabetes [20]. Rosenthal et al. reported from 30 diabetic patients who underwent LSG; the HbA1c had decreased postoperatively to 6.02% ± 0.57% (n = 11) at the second month and 5.9% ± 0.33% (n = 12) at the sixth month [25].

FBG: A meta-analysis study on 914 T2DM patients was conducted to study the significance of preoperative FBG level and the remission of diabetes in obese patients. They found that in non-Asian patients, the remission rate may be affected by FBG. Patients with increased levels of FBG will possibly have lower rates of diabetes remission [20]. Blackstone et al. reported that patients who had lower FBG and HbA1c levels achieved diabetes remission despite preoperative insulin use [17]. This was similarly stated by Robert et al., who concluded that despite the type of bariatric procedure, preoperative FBG < 114 mg/dL and HbA1c < 7.1% are predictors for the remission of T2DM at one-year follow-up [28].

The DiaRem score system: Multiple scoring systems have been published to predict the possibility of diabetes remission. However, not all scoring systems can be used to predict diabetes remission across all populations. The DiaRem score system was developed by Dr. Still in 2014. It was proposed to predict diabetes remission (from 2% to 99%) after RYGB. This scoring system comprises four preoperative predictors (age, HbA1c, use of insulin, and antidiabetic medication). The score ranges from 0 to 22, where higher scores indicate a decreased remission rate [29]. The parameters of the scoring system are age, HbA1c, use of antidiabetic medications, and insulin use. The points for patient age are assigned as follows: 0 points if the patient age was less than 40 years old, 1 point if the age was ranging from 40 to 49 years old, 2 points if the age ranges from 50 to 59 years old, and 3 points if the age of the patient was 60 years old or more. For the HbA1c, the points are assigned as follows: 0, 2, 4, and 6 if the HbA1c values were less than 6.5, 6.5-6.9, 7-8.9, and more than 9, correspondingly. As for the use of the antidiabetic medications, 0 points are given if the patient was on metformin only, whereas 3 points are given if the patient was on sulfonylurea or insulin sensitizers other than metformin. Lastly, if the patient was using insulin for controlling blood sugar, the point assigned is 0, whereas 10 points are given if the patient was not using insulin [29] (see Appendix Table).

## Materials and methods

This was a non-randomized controlled trial conducted at King Fahad Specialist Hospital in Buraydah, Saudi Arabia. We obtained ethical approval from the Qassim Region Research Ethics Committee (QREC) to conduct this hospital-based research on November 3, 2021 (number: 1443-598215). We have ensured the protection and anonymity of research participants’ privacy. All research conductors had completed the certification course from the National Committee of Bioethics (NCBE) at King Abdulaziz City of Science and Technology.

Visiting patients at first screening were enrolled based on eligibility criteria. Inclusion criteria were all morbidly obese patients diagnosed with T2DM who are scheduled to undergo bariatric surgery. The study’s population was estimated to be around 134 patients. Our sample size was calculated based on preset values, which were entered in the famous sample calculation software G-power application, resulting in a needed sample size of approximately 96 subjects. Data were collected according to the needed parameters such as gender, age, BMI, duration of DM, medications (insulin, oral antihyperglycemic agents, number of tablets if used, or no medications use), presence of comorbidities, such as hypertension and dyslipidemia, HbA1c level (before surgery and at third, sixth, and 12th months after surgery), and FBG level (before and after the surgery). Another section in the collection form regarding the surgery includes the type of surgery performed and the date of surgery. We then contacted each participant of our study sample in person to obtain the missing data at their medical files to be recorded on separate data sheets (Google Forms) for each individual.

Statistical analysis

Descriptive statistics were presented using numbers, percentages, means, and standard deviations. The association between the DiaRem score and the characteristics of the DM patients before and after bariatric surgery had been performed using Fischer's exact test and one-way analysis of variance (ANOVA) test, respectively. A p-value cut-off point of 0.05 at 95% CI was used to determine statistical significance. All data analyses were performed using Statistical Packages for Software Sciences (SPSS) version 26 (IBM Corp., Armonk, NY).

## Results

**Figure 1:**
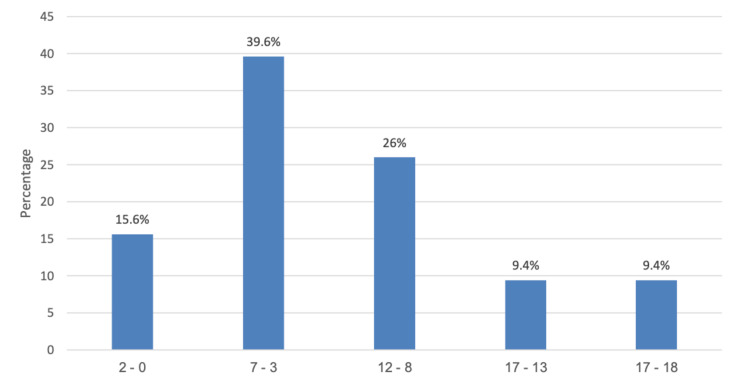
DiaRem results according to the group

**Figure 2:**
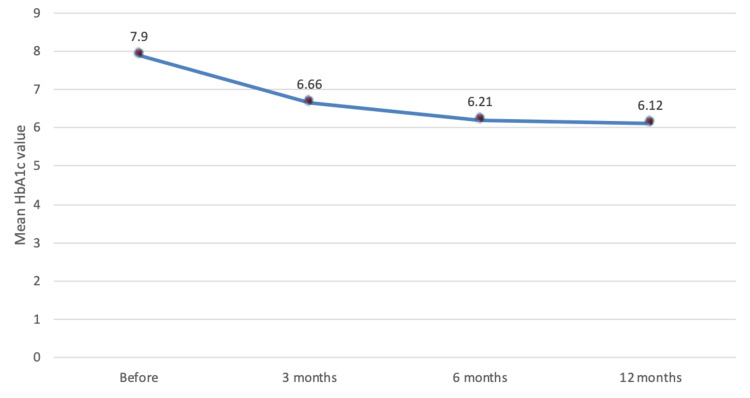
The trend of mean HbA1c value before and 12 months after bariatric surgery HbA1c, Glycated hemoglobin.

We reviewed 96 diabetic patients who underwent bariatric surgery. As seen in Table 1, the mean age of the patients was 46.5 (SD 9.09), with nearly two-thirds being females (63.5%). The majority of the patients started DM medication immediately after the diagnosis (77.1%). The most commonly associated comorbidity was hypertension (50%) and hypothyroidism (14.6%), while the most dominant type of surgery was lap sleeve gastrectomy (96.9%). Half (50%) of the patients experienced hypoglycemia, while 55.2% of the patients stopped the medication. Furthermore, 28.1% reported that they stopped medication immediately after the surgery. Based on the given criteria, we found that the majority of patients had a DiaRem score of 3-7, which accounts for 39.6% of our study sample, 26% had 8-12 points, while 15.6% of patients had a score of 0-2 (Figure 1). The mean BMI value was 43.1 kg/m^2^, while the mean duration of DM was 10.4 years. In addition, the mean values of HbA1c at baseline and third, sixth, and 12 months after surgery were 7.90, 6.66, 6.21, and 6.12, respectively (Figure 2). The mean values of FBG before and after surgery were 11.4 and 6.34, respectively.

**Table 1:** Baseline characteristics of the DM patients who underwent bariatric surgery (n = 96) *Variable with multiple response answers. DM, Diabetes mellitus; SD, standard deviation; BMI, body mass index; HbA1c, glycated hemoglobin; FBG, fasting blood glucose.

Variables	Mean (%)
Age in years (mean ± SD)	46.5 ± 9.09
Gender	
Male	35 (36.5%)
Female	61 (63.5%)
Time since start medication	
Since diagnosis	74 (77.1%)
Less than a year after diagnosis	11 (11.5%)
One year or more after diagnosis	11 (11.5%)
Associated comorbidity*	
Hypertension	48 (50.0%)
Hypothyroidism	14 (14.6%)
Bronchial asthma	10 (10.4%)
Rheumatoid arthritis	07 (07.3%)
Others	08 (08.3%)
None	38 (39.6%)
Type of surgery	
Lap sleeve gastrectomy	93 (96.9%)
Lap gastric mini bypass	02 (02.1%)
Lap Roux-en-Y gastric bypass	01 (01.0%)
Experienced hypoglycemia	
Yes	48 (50.0%)
No	48 (50.0%)
Stopped medications	
Yes	53 (55.2%)
No	43 (44.8%)
Interval duration between surgery and stopping medications	
Immediately after the surgery	27 (28.1%)
Three months after the surgery	10 (10.4%)
More than three months after the surgery	16 (16.7%)
Did not stop medications	43 (44.8%)
DiaRem results	
0–2	15 (15.6%)
3–7	38 (39.6%)
8–12	25 (26.0%)
13–17	09 (09.4%)
18-22	09 (09.4%)
	Mean ± SD
BMI kg/m^2^	43.1 ± 6.78
Duration of DM in years	10.4 ± 6.67
Pre-HbA1c %	7.90 ± 1.99
HbA1c % after three months	6.66 ± 1.34
HbA1c % after six months	6.21 ± 1.19
HbA1c % after 12 months	6.12 ± 1.07
Pre-FBG in mmol/L	11.4 ± 17.1
Post-FBG in mmol/L	6.34 ± 2.33

Table 2 presented the relationship between the DiaReam score and the characteristics of the patients with diabetes before and after bariatric surgery. The lowest DiaRem scores (0-2) were associated with a shorter duration of DM (p < 0.001), lower mean values of post-FBG (p < 0.001), and HbA1c at baseline (p < 0.001) at three months (p < 0.001), six months (p < 0.001), and 12 months (p = 0.043). The mean BMI value was significantly lower in the DiaRem 18-22 score groups (p = 0.003). On the other hand, the relationship between DiaRem score groups among gender, associated comorbidity, the incidence of hypoglycemia, discontinuation of medication, age in years, and pre-FBG were not statistically significant (p > 0.05).

**Table 2:** Association between the DiaRem score and the characteristics of the DM patients before and after bariatric surgery (n = 96) Values are given as n(%) or mean ± SD. ^§^P-value has been calculated using Fischer’s exact test. ^‡^P-value has been calculated using a one-way ANOVA test. **Significant at p < 0.05 level. DM, Diabetes mellitus; SD, standard deviation; BMI, body mass index; HbA1c, glycated hemoglobin; FBG, fasting blood glucose.

Factor	DiaRem Score					Exact Test	P-Value^§^
	0–2 N (%)	3–7 N (%)	8–12 N (%)	13–17 N (%)	18–22 N (%)		
Gender							
Male	02 (13.3%)	16 (42.1%)	11 (44.0%)	02 (22.2%)	04 (44.4%)	5.694	0.214
Female	13 (86.7%)	22 (57.9%)	14 (56.0%)	07 (77.8%)	05 (55.6%)		
Associated comorbidity							
Yes	12 (80.0%)	19 (50.0%)	16 (64.0%)	05 (55.6%)	06 (66.7%)	4.475	0.345
No	03 (20.0%)	19 (50.0%)	09 (36.0%)	04 (44.4%)	03 (33.3%)		
Hypoglycemia							
Yes	07 (46.7%)	19 (50.0%)	10 (40.0%)	08 (88.9%)	04 (44.4%)	6.679	0.151
No	08 (53.3%)	19 (50.0%)	15 (60.0%)	01 (11.1%)	05 (55.6%)		
Stop medication							
Yes	12 (80.0%)	19 (50.0%)	14 (56.0%)	03 (33.3%)	05 (55.6%)	5.901	0.207
No	03 (20.0%)	19 (50.0%)	11 (44.0%)	06 (66.7%)	04 (44.4%)		
	Mean ± SD	Mean ± SD	Mean ± SD	Mean ± SD	Mean ± SD	F-test	P-value^‡^
Age in years	41.2 ± 7.00	47.2 ± 9.31	46.9 ± 10.3	46.2 ± 7.34	51.1 ± 6.49	2.008	0.100
BMI kg/m^2^	45.7 ± 7.24	41.3 ± 4.95	44.4 ± 6.59	47.9 ± 10.0	38.1 ± 4.84	4.307	0.003**
Duration of DM in years	7.13 ± 3.66	8.63 ± 5.15	12.6 ± 6.97	10.6 ± 7.26	17.6 ± 0.92	5.809	<0.001**
Pre-HbA1c%	5.74 ± 0.46	7.69 ± 2.02	8.71 ± 1.56	8.06 ± 1.49	9.94 ± 1.52	11.455	<0.001**
HbA1c% after three months	5.40 ± 0.71	6.54 ± 1.22	7.01 ± 1.51	6.58 ± 0.93	7.84 ± 1.02	5.676	<0.001**
HbA1c% after six months	5.35 ± 0.47	6.24 ± 1.23	6.60 ± 1.34	6.38 ± 1.60	6.70 ± 0.42	2.618	0.043**
HbA1c% after 12 months	5.42 ± 0.66	6.07 ± 1.17	6.32 ± 1.15	6.07 ± 0.46	7.04 ± 0.59	3.185	0.018**
Pre-FBG in mmol/L	18.5 ± 43.7	9.09 ± 4.28	10.7 ± 4.47	10.1 ± 3.90	13.2 ± 3.69	0.814	0.519
Post-FBG in mmol/L	4.92 ± 0.79	6.06 ± 1.95	6.83 ± 2.79	5.86 ± 1.09	9.09 ± 2.76	6.153	<0.001**

## Discussion

The present study attempted to establish the sensitivity of the DiaRem scoring system in forecasting the resolution of T2DM in morbidly obese patients who underwent bariatric surgery at King Fahad Specialist Hospital in Buraydah, Saudi Arabia. Using the DiaRem scoring criteria published by Still et al. [29], a calculated score of 3-7 points was seen in 40% of patients, 8-12 points in 26% of patients, and 0-2 points in 15.6% of patients.

Moreover, 9.6% of patients had a score ranging from 13 to 17, and an estimated score of 18-22 was observed in the same percentage of the study sample. In a study by Wood et al. [30], nearly all patients (84%) who underwent Roux-en-Y gastric bypass (RYGB) had a DiaRem score of 0-2 points, which was higher than our report. These authors further emphasized that the largest proportion of patients to achieve remission was in patients with a DiaRem score of 0-2, with a sheer drop-off in remission for scores above 7, adding that 73% of LSG patients and 57% of laparoscopic adjustable gastric banding (LAGB) patients experienced remission. This is comparable to the paper of Panunzi et al. [24], indicating that 64% of surgical patients and 15% in the medical arm experienced DM remission. The authors further detailed that gastric-only patients yielded 60% remission after bariatric surgery, whereas gastric plus diversion patients yielded 76% remission. These indicate that the advantage of an early operation together with better-controlled glycemia could be a therapeutic option for patients with T2DM.

The DiaRem scoring criteria suggest that the higher the score, the lower the chance for diabetes remission, while the lower the score, the higher the chance of diabetes remission. Accordingly, we noted that the patients who had a longer duration of DM had significantly higher DiaRem scores, whereas lower DiaRem scores were significantly associated with a shorter duration of DM (p < 0.001). This report is consistent with the study of English et al. [14]. Based on their investigation, these authors found that shorter duration of DM and non-insulin use were significant predictors of remission, with patients not requiring insulin attaining remission rates of 92% while the rates are only 40% for those who were using insulin. Another paper conducted by Panunzi et al. [24] documented that patients with a shorter duration of DM before surgery independently predicted higher remission rates, which was also true in our study.

The literature suggests that lower mean HbA1c values were associated with diabetes remission. This is consistent with our reports as patients with lower HbA1c were significantly associated with lower DiaRem scores (p < 0.001). Consistently, English et al. [14] reported that optimal control of glucose preoperatively indicates that when patients decreased their HbA1c before surgery by 1%, they were 68% more likely to remit. Another study done in the United States [29] reported that early and late diabetes remission correlated with lower HbA1c levels.

A study done in the United States [14] indicated that optimizing glucose prior to RYGB was beneficial among DM patients. They are more likely to achieve DM remission one year after the surgery. This was similarly detected in our study. Our study found that lower HbA1c was associated with higher chances of diabetic remission. Conversely, data in this study also demonstrated that a lower mean BMI value was associated with decreased diabetes remission rates.

Still et al. [29] documented that younger age was associated with early and late DM remission rates. This is not true in our study as we found that there was no difference between the DiaRem scores among age, gender, associated comorbidity, the incidence of hypoglycemia, and medication discontinuation, which was consistent with the paper of Blackstone et al. [17]; age, race, and gender were not relevant factors of remission.

Moreover, in this study, we found that the mean FBG values after the surgery were significantly lower than the baseline (p = 0.006). This was similar to that of Hamza et al. [22]. They reported that the mean FBG and glycosylated hemoglobin levels decreased to normal range postoperatively due to the bariatric procedure. This indicates that the bariatric procedure is one of the most effective interventions for treating and managing patients with T2DM.

Even with these findings, this study has its limitations. First, some participants’ data at three, six, and 12 months were unavailable on their medical records, although we managed to reach a total of 96 patients, which is our original sample size. When we implemented virtual appointments to contact patients with missed data, we found out that many of them had lost their follow-up due to the COVID-19 pandemic as many appointments were originally scheduled during the quarantine time; this can reduce the accuracy of detecting the resolution of their DM.

Furthermore, a calculated sample size of 96 subjects might be considered insufficient regarding the increasing rate of bariatric surgeries done for morbidly obese diabetic patients. Hence, the authors recommend implanting more related research studies in different regions of Saudi Arabia to make even more fair judgments about the resolution of type two diabetes after bariatric surgery.

## Conclusions

Consistent with the literature, we found that having increased BMI, shorter duration of DM, lower values of HbA1c, and post-FBG were associated with a greater chance of diabetes remission postoperatively. We emphasize the importance of using accurate benchmarks to distinguish remission and concentrate on metabolic health improvements to help physicians choose the best surgical option to treat and manage T2DM, thus providing more genuine patient expectations.

## Appendices

**Table 3:** The DiaRem score system HbA1c, Glycated hemoglobin.

Punctuation	Parameters
Age	
0	Less than 40
1	40-49
2	50-59
3	60 or more
HbA1C	
0	Less than 6.5
2	6.5–6.9
4	7–8.9
6	More than 9
Use of antidiabetic medications	
0	Metformin only
3	Sulfonylurea and insulin sensitizers other than metformin
Insulin use	
0	Yes
10	No

Abbreviations

DM, diabetes mellitus; BMI, body mass index; T2DM, type 2 diabetes mellitus; HbA1c, glycated hemoglobin; FBG, fasting blood glucose; RYGB, Roux-en-Y gastric bypass; LSG, laparoscopic sleeve gastrectomy; ANOVA, analysis of variance; CI, confidence interval; SD, standard deviation.
